# Supplementary material for: Tuberculosis treatment intervention trials in Africa: A cross-sectional bibliographic study and spatial analysis
Source: PLoS One. 2021 Mar 19;16(3):e0248621. doi: 10.1371/journal.pone.0248621 (PMC7978376; doi:10.1371/journal.pone.0248621)
Supplement: S1 Table — (DOCX) [file pone.0248621.s001.docx]

## **S1 Table. Excluded studies**

| Study | Reason for exclusion |
| --- | --- |
| Amagon et al., 2017 | Study design |
| Batbold et al., 2017 | Not conducted in Africa |
| Biddulph, 1990 | Study design |
| Bouton et al., 2017 | Study design |
| Chabala et al., 2018 | Study design |
| Chaulet et al., 1967 | Study design |
| Chum et al., 1995 | Study design |
| Conde et al., 2014 | Not conducted in Africa |
| Cresswell et al., 2018 | Study design |
| Davis et al., 2019 | Population |
| Dosumu, 2002 | Study design |
| Elsawaf, 2013 | Not conducted in Africa |
| Fairall et al., 2005 | No intervention |
| Fredlund, 1990 | Study design |
| Galarza et al., 1995 | Not conducted in Africa |
| Gninafon et al., 1995 | Study design |
| Grant et al., 2010 | No intervention |
| Grosset and Ammerman, 2013 | Study design |
| Hayes-Larson et al., 2017 | population |
| Jayakumar et al., 2015 | No intervention |
| Kufa et al., 2018 | Population |
| Lopez-Cortes et al., 2002 | Not conducted in Africa |
| Montane et al., 2017 | Not conducted in Africa |
| Moriarty et al., 2019 | Study design |
| Moro et al., 2017 | Not conducted in Africa |
| Moultrie et al., 2015 | Study design |
| Naidoo et al., 2017 | Study design |
| Ndishimye et al., 2015 | Study design |
| Peter et al., 2016 | No intervention |
| Pym et al., 2016 | Study design |
| Ritchie et al., 2016 | Study design |
| Salieh et al., 2005 | Study design |
| Sanchez-Albisua et al., 1997 | Study design |
| Schull et al., 2010 | Study design |
| Seifart et al., 1995 | Study design |
| Sharifi-Rad et al., 2017 | Study design |
| Song et al., 2015 | Not conducted in Africa |
| Stek et al., 2016 | Study design |
| Sumari-de Boer et al., 2019 | Study design |
| Torres et al., 2015 | Not conducted in Africa |
| Tupasi et al., 2016 | Study design |
| Unknown, 1976 | Study design |
| Unknown, 1971 | Study design |
| Unknown, 1972 | Study design |
| Wagstaff et al., 2019 | Population |
| Zent and Smith, 1995 | Study design |

**References**

1. Amagon KI, Awodele O, Akindele AJ. Methionine and vitamin B‐complex ameliorate antitubercular drugs‐induced toxicity in exposed patients. Pharmacology research & perspectives. 2017;5(5):e00360.

2. Batbold U, Butov DO, Kutsyna GA, Damdinpurev N, Grinishina EA, Mijiddorj O, et al. Double-blind, placebo-controlled, 1:1 randomized Phase III clinical trial of Immunoxel honey lozenges as an adjunct immunotherapy in 269 patients with pulmonary tuberculosis. Immunotherapy. 2017;9(1):13-24. Epub 2016/11/22. doi: 10.2217/imt-2016-0079. PubMed PMID: 27868466.

3. Biddulph J. Short course chemotherapy for childhood tuberculosis. Pediatr Infect Dis J. 1990;9(11):794-801. Epub 1990/11/01. PubMed PMID: 2263427.

4. Bouton TC, Phillips PP, Mitnick CD, Peloquin CA, Eisenach K, Patientia RF, et al. An optimized background regimen design to evaluate the contribution of levofloxacin to multidrug-resistant tuberculosis treatment regimens: study protocol for a randomized controlled trial. Trials. 2017;18(1):1-8.

5. Chabala C, Turkova A, Thomason MJ, Wobudeya E, Hissar S, Mave V, et al. Shorter treatment for minimal tuberculosis (TB) in children (SHINE): a study protocol for a randomised controlled trial. Trials. 2018;19(1):1-12.

6. Chaulet P, Larbaoui D, Grosset J, Abderrahim K. Intermittent chemotherapy with isoniazid and streptomycin in Algiers. Tubercle. 1967;48(2):128-36. Epub 1967/06/01. PubMed PMID: 6058030.

7. Chum HJ, Ilmolelian G, Rieder HL, Msangi J, Mwinyi N, Zwahlen M, et al. Impact of the change from an injectable to a fully oral regimen on patient adherence to ambulatory tuberculosis treatment in Dar es Salaam, Tanzania. Tubercle and lung disease [Internet]. 1995; 76(4):[286-9 pp.]. Available from: http://onlinelibrary.wiley.com/o/cochrane/clcentral/articles/923/CN-00670923/frame.html.

8. Conde MB, Cavalcante SC, Dalcolmo M, Mello F, Duarte R, Loredo C, et al. A Phase 2 Trial of a Rifapentine Plus Moxifloxacin-Based Regimen for Pulmonary TB Treatment. Topics in Antiviral Medicine. 2014;22(e-1):47.

9. Cresswell FV, Ssebambulidde K, Grint D, Te Brake L, Musabire A, Atherton RR, et al. High dose oral and intravenous rifampicin for improved survival from adult tuberculous meningitis: a phase II open-label randomised controlled trial (the RifT study). Wellcome Open Res. 2018;3:83. Epub 2018/09/04. doi: 10.12688/wellcomeopenres.14691.1. PubMed PMID: 30175245; PubMed Central PMCID: PMCPMC6113880.

10. Davis JL, Turimumahoro P, Meyer AJ, Ayakaka I, Ochom E, Ggita J, et al. Home-based tuberculosis contact investigation in Uganda: a household randomised trial. ERJ Open Res. 2019;5(3). Epub 2019/08/02. doi: 10.1183/23120541.00112-2019. PubMed PMID: 31367636; PubMed Central PMCID: PMCPMC6661318.

11. Dosumu EA. Side-effects of drugs used in directly observed treatment short-course in newly diagnosed pulmonary tuberculosis subjects in Nigerians: a controlled clinical study. Nigerian postgraduate medical journal [Internet]. 2002; 9(1):[34-7 pp.]. Available from: http://onlinelibrary.wiley.com/o/cochrane/clcentral/articles/357/CN-00379357/frame.html.

12. Elsawaf A. Outcome of surgical versus conservative management of cervical spine myelopathy secondary to cervical tuberculosis. Neurosurg Rev. 2013;36(4):621-8; discussion 8. Epub 2013/05/29. doi: 10.1007/s10143-013-0475-9. PubMed PMID: 23712475.

13. Fairall LR, Zwarenstein M, Bateman ED, Bachmann M, Lombard C, Majara BP, et al. Effect of educational outreach to nurses on tuberculosis case detection and primary care of respiratory illness: pragmatic cluster randomised controlled trial. BMJ. 2005;331(7519):750-4. doi: 10.1136/bmj.331.7519.750. PubMed PMID: 16195293; PubMed Central PMCID: PMC1239979.

14. Fredlund VG. Six-month intermittent chemotherapy for tuberculosis in the Mseleni Health Ward of KwaZulu. S Afr Med J. 1990;77(8):405-7. Epub 1990/04/21. PubMed PMID: 2330525.

15. Galarza I, Canete C, Granados A, Estopa R, Manresa F. Randomised trial of corticosteroids in the treatment of tuberculous pleurisy. Thorax. 1995;50(12):1305-7. Epub 1995/12/01. PubMed PMID: 8553306; PubMed Central PMCID: PMCPMC1021356.

16. Gninafon M, Lambregts-van Weezenbeek CS, Tawo L, Trebucq A. Ethambutol versus streptomycin during the hospitalized intensive phase of tuberculosis treatment in Benin. Tuber Lung Dis. 1995;76(4):373-4. Epub 1995/08/01. PubMed PMID: 7579325.

17. Grant AD, Mngadi KT, van Halsema CL, Luttig MM, Fielding KL, Churchyard GJ. Adverse events with isoniazid preventive therapy: experience from a large trial. AIDS. 2010;24 Suppl 5:S29-36. Epub 2010/11/26. doi: 10.1097/01.aids.0000391019.10661.66. PubMed PMID: 21079425.

18. Grosset JH, Ammerman NC. Dose-ranging activity of the newly registered antituberculosis drug bedaquiline (TMC207). Expert Review of Anti-Infective Therapy. 2013;11(7):649-51.

19. Hayes-Larson E, Hirsch-Moverman Y, Saito S, Frederix K, Pitt B, Maama BL, et al. Prevalence, patterns, and correlates of HIV disclosure among TB-HIV patients initiating antiretroviral therapy in Lesotho. AIDS care. 2017;29(8):978-84. Epub 2017/01/20. doi: 10.1080/09540121.2017.1280124. PubMed PMID: 28100068; PubMed Central PMCID: PMCPMC5469711.

20. Jayakumar A, Vittinghoff E, Segal M, MacKenzie W, Johnson J, Gitta P, et al. Serum biomarkers of treatment response within a randomized clinical trial for pulmonary tuberculosis. Tuberculosis (edinburgh, scotland) [Internet]. 2015; 95(4):[415-20 pp.]. Available from: http://onlinelibrary.wiley.com/o/cochrane/clcentral/articles/139/CN-01102139/frame.html

21. Kufa T, Fielding KL, Hippner P, Kielmann K, Vassall A, Churchyard GJ, et al. An intervention to optimise the delivery of integrated tuberculosis and HIV services at primary care clinics: results of the MERGE cluster randomised trial. Contemp Clin Trials. 2018;72:43-52. Epub 2018/07/28. doi: 10.1016/j.cct.2018.07.013. PubMed PMID: 30053431.

22. Lopez-Cortes LF, Ruiz-Valderas R, Viciana P, Alarcon-Gonzalez A, Gomez-Mateos J, Leon-Jimenez E, et al. Pharmacokinetic interactions between efavirenz and rifampicin in HIV-infected patients with tuberculosis. Clin Pharmacokinet. 2002;41(9):681-90. Epub 2002/07/20. PubMed PMID: 12126459.

23. Montane E, Barriocanal AM, Arellano AL, Valderrama A, Sanz Y, Perez-Alvarez N, et al. Pilot, double-blind, randomized, placebo-controlled clinical trial of the supplement food Nyaditum resae(R) in adults with or without latent TB infection: Safety and immunogenicity. PLoS One. 2017;12(2):e0171294. Epub 2017/02/10. doi: 10.1371/journal.pone.0171294. PubMed PMID: 28182700; PubMed Central PMCID: PMCPMC5300153.

24. Moriarty AS, Louwagie GM, Mdege ND, Morojele N, Tumbo J, Omole OB, et al. ImPROving TB outcomes by modifying LIFE-style behaviours through a brief motivational intervention followed by short text messages (ProLife): study protocol for a randomised controlled trial. Trials. 2019;20(1):457. Epub 2019/07/28. doi: 10.1186/s13063-019-3551-9. PubMed PMID: 31349850; PubMed Central PMCID: PMCPMC6660690.

25. Moro RN, Sterling TR, Saukkonen J, Vernon A, Horsburgh CR, Chaisson RE, et al. Factors associated with non-completion of follow-up: 33-month latent tuberculous infection treatment trial. The international journal of tuberculosis and lung disease : the official journal of the International Union against Tuberculosis and Lung Disease. 2017;21(3):286-96. Epub 2017/01/15. doi: 10.5588/ijtld.16.0469. PubMed PMID: 28087928.

26. Moultrie H, McIlleron H, Sawry S, Kellermann T, Wiesner L, Kindra G, et al. Pharmacokinetics and safety of rifabutin in young HIV-infected children receiving rifabutin and lopinavir/ritonavir. The Journal of antimicrobial chemotherapy. 2015;70(2):543-9. Epub 2014/10/05. doi: 10.1093/jac/dku382. PubMed PMID: 25281400; PubMed Central PMCID: PMCPMC4291235.

27. Naidoo K, Gengiah S, Yende-Zuma N, Padayatchi N, Barker P, Nunn A, et al. Addressing challenges in scaling up TB and HIV treatment integration in rural primary healthcare clinics in South Africa (SUTHI): a cluster randomized controlled trial protocol. Implementation Science. 2017;12(1):129.

28. Ndishimye P, Seghrouchni F, Domokos B, Soritau O, Sadak A, Homorodean D, et al. Evaluation of interleukin-10 levels in the plasma of patients with various stages of tuberculosis. Clujul medical (1957). 2015;88(2):164-7. Epub 2015/11/04. doi: 10.15386/cjmed-459. PubMed PMID: 26528066; PubMed Central PMCID: PMCPMC4576778.

29. Peter J, Zijenah L, Chanda D, Clowes P, Lesosky M, Gina P, et al. Effect on mortality of point-of-care, urine-based lipoarabinomannan testing to guide tuberculosis treatment initiation in HIV-positive hospital inpatients: a pragmatic, parallel-group, multicountry, open-label, randomised controlled trial. Lancet (london, england) [Internet]. 2016; 387(10024):[1187-97 pp.]. Available from: http://onlinelibrary.wiley.com/o/cochrane/clcentral/articles/589/CN-01141589/frame.html

30. Pym AS, Diacon AH, Tang SJ, Conradie F, Danilovits M, Chuchottaworn C, et al. Bedaquiline in the treatment of multidrug- and extensively drug-resistant tuberculosis. The European respiratory journal. 2016;47(2):564-74. Epub 2015/12/10. doi: 10.1183/13993003.00724-2015. PubMed PMID: 26647431.

31. Ritchie LMP, van Lettow M, Makwakwa A, Chan AK, Hamid JS, Kawonga H, et al. The impact of a knowledge translation intervention employing educational outreach and a point-of-care reminder tool vs standard lay health worker training on tuberculosis treatment completion rates: study protocol for a cluster randomized controlled trial. Trials. 2016;17(1):439.

32. Salieh M, Elhaj H, Adam K, Hassan A, Slama K, Enarson DA. Brief smoking cessation intervention with tuberculosis patients in Sudan. Society for Research on Nicotine and Tobacco 11th Annual Meeting, 20-23 March 2005; Prague, Czech Republic [Internet]. 2005; 7(4):[686- pp.]. Available from: http://onlinelibrary.wiley.com/o/cochrane/clcentral/articles/420/CN-00527420/frame.html.

33. Sanchez-Albisua I, Vidal ML, Joya-Verde G, del Castillo F, de Jose MI, Garcia-Hortelano J. Tolerance of pyrazinamide in short course chemotherapy for pulmonary tuberculosis in children. Pediatr Infect Dis J. 1997;16(8):760-3. Epub 1997/08/01. PubMed PMID: 9271037.

34. Schull MJ, Banda H, Kathyola D, Fairall L, Martiniuk A, Burciul B, et al. Strengthening health human resources and improving clinical outcomes through an integrated guideline and educational outreach in resource-poor settings: a cluster-randomized trial. Trials. 2010;11:118. Epub 2010/12/07. doi: 10.1186/1745-6215-11-118. PubMed PMID: 21129211; PubMed Central PMCID: PMCPMC3017521.

35. Seifart HI, Donald PR, de Villiers JN, Parkin DP, Jaarsveld PP. Isoniazid elimination kinetics in children with protein-energy malnutrition treated for tuberculous meningitis with a four-component antimicrobial regimen. Ann Trop Paediatr. 1995;15(3):249-54. Epub 1995/09/01. PubMed PMID: 8534045.

36. Sharifi-Rad J, Salehi B, Stojanovic-Radic ZZ, Fokou PVT, Sharifi-Rad M, Mahady GB, et al. Medicinal plants used in the treatment of tuberculosis - Ethnobotanical and ethnopharmacological approaches. Biotechnology advances. 2017. Epub 2017/07/12. doi: 10.1016/j.biotechadv.2017.07.001. PubMed PMID: 28694178.

37. Song T, Lee M, Jeon H-S, Park Y, Dodd LE, Dartois V, et al. Linezolid trough concentrations correlate with mitochondrial toxicity-related adverse events in the treatment of chronic extensively drug-resistant tuberculosis. EBioMedicine. 2015;2(11):1627-33.

38. Stek C, Schutz C, Blumenthal L, Thienemann F, Buyze J, Nöstlinger C, et al. Preventing paradoxical tuberculosis-associated immune reconstitution inflammatory syndrome in high-risk patients: protocol of a randomized placebo-controlled trial of prednisone (PredART Trial). JMIR research protocols. 2016;5(3):e173.

39. Sumari-de Boer M, Pima FM, Ngowi KM, Chelangwa GM, Mtesha BA, Minja LM, et al. Implementation and effectiveness of evriMED with short messages service (SMS) reminders and tailored feedback compared to standard care on adherence to treatment among tuberculosis patients in Kilimanjaro, Tanzania: proposal for a cluster randomized controlled trial. Trials. 2019;20(1):426. Epub 2019/07/14. doi: 10.1186/s13063-019-3483-4. PubMed PMID: 31300028; PubMed Central PMCID: PMCPMC6626331.

40. Torres M, Garcia-Garcia L, Cruz-Hervert P, Guio H, Carranza C, Ferreyra-Reyes L, et al. Effect of isoniazid on antigen-specific interferon-(gamma) secretion in latent tuberculosis. European Respiratory Journal. 2015;45(2):473-82.

41. Tupasi T, Gupta R, Danilovits M, Cirule A, Sanchez-Garavito E, Xiao H, et al. Building clinical trial capacity to develop a new treatment for multidrug-resistant tuberculosis. Bulletin of the world health organization [Internet]. 2016; 94(2):[147-52 pp.]. Available from: http://onlinelibrary.wiley.com/o/cochrane/clcentral/articles/592/CN-01307592/frame.html.

42. Unknown. First-line chemotherapy in the retreatment of bacteriological relapses of pulmonary tuberculosis following a shortcourse regimen. Lancet. 1976;1(7952):162-3. Epub 1976/01/24. PubMed PMID: 54680.

43. Unknown. Streptomycin plus PAS plus pyrazinamide in the retreatment of pulmonary tuberxulisis in East Africa. Tubercle. 1971;52(3):191-8. Epub 1971/09/01. PubMed PMID: 4938216.

44. Unknown. Streptomycin plus pas plus pyrazinamide in the retreatment of pulmonary tuberculosis in East Africa. East Afr Med J. 1972;49(1):7-15. Epub 1972/01/01. PubMed PMID: 4555041.

45. Wagstaff A, van Doorslaer E, Burger R. SMS nudges as a tool to reduce tuberculosis treatment delay and pretreatment loss to follow-up. A randomized controlled trial. PloS one. 2019;14 (6) (no pagination)(e0218527). doi: 10.1371/journal.pone.0218527. PubMed PMID: CN-01954048.

46. Zent C, Smith P. Study of the effect of concomitant food on the bioavailability of rifampicin, isoniazid and pyrazinamide. Tubercle and lung disease [Internet]. 1995; 76(2):[109-13 pp.]. Available from: http://onlinelibrary.wiley.com/o/cochrane/clcentral/articles/967/CN-00114967/frame.html.
