# Supplementary material for: Tuberculosis treatment intervention trials in Africa: A cross-sectional bibliographic study and spatial analysis
Source: PLoS One. 2021 Mar 19;16(3):e0248621. doi: 10.1371/journal.pone.0248621 (PMC7978376; doi:10.1371/journal.pone.0248621)
Supplement: S2 Table — (DOCX) [file pone.0248621.s002.docx]

## **S2 Table. Trial Funders**

|  | Number of trials funded | Percent |
| --- | --- | --- |
| Not reported | 35 | 20.00 |
| International government | 29 | 16.57 |
| Pharmaceutical | 17 | 9.71 |
| International government and International non-government | 14 | 8 |
| International non-government | 10 | 5.71 |
| International government and Academic | 9 | 5.14 |
| Local government, International non-government, International government | 6 | 3.43 |
| International non-government, International government, Academic | 6 | 3.43 |
| Academic | 5 | 2.86 |
| Local government | 4 | 2.29 |
| Pharmaceutical and Local government | 4 | 2.29 |
| Local government and Academic | 4 | 2.29 |
| International non-government/Local government | 3 | 1.71 |
| Pharmaceutical/International non-government | 3 | 1.71 |
| International non-government/Local non-government/Academic | 3 | 1.71 |
| Local government /International non-government/Local non-government | 3 | 1.71 |
| International government /Local government/academic | 3 | 1.71 |
| Pharmaceutical/ International Local government /International non-government | 2 | 1.14 |
| Local non-government | 2 | 1.14 |
| None Received | 2 | 1.14 |
| International non-government/Academic | 2 | 1.14 |
| International government /Pharmaceutical | 2 | 1.14 |
| Local non-government/Local government/Academic | 2 | 1.14 |
| Local government/ International government | 1 | 0.57 |
| Local government/International non-government/International government/Local non-government | 1 | 0.57 |
| International government/Local non-government | 1 | 0.57 |
| International government / Pharmaceutical | 1 | 0.57 |
| International government /Pharmaceutical/Academic | 1 | 0.57 |
|  |  |  |
| Total | 175 | 100 |
