# Supplementary material for: Tuberculosis treatment intervention trials in Africa: A cross-sectional bibliographic study and spatial analysis
Source: PLoS One. 2021 Mar 19;16(3):e0248621. doi: 10.1371/journal.pone.0248621 (PMC7978376; doi:10.1371/journal.pone.0248621)
Supplement: Attachment — Submitted filename: Response to Reviewers.docx [file pone.0248621.s004.docx]

Cochrane South Africa

South African Medical Research Council

Francie van Zijl Drive, Parow Valley

Cape Town, 7501

South Africa

01 December 2020

**To: The Editor**

**PLOS ONE**

Dear Editor

Thank you for the feedback. We would like to resubmit the attached manuscript, titled **“Tuberculosis treatment intervention trials in Africa: a cross-sectional bibliographic study and spatial analysis”** after addressing reviewers’ comments. Please see below a table of the reviewers’ comments and our responses.

| **Reviewers’ comments** | **Authors’ response** |
| --- | --- |
| **Reviewer 1:** |  |
| **Background** |  |
| Background does not indicate what is known and the gap (unknown) | Thank you.  To address this more clearly, we have added a paragraph to the background as follows:  “It is well known that HIV/AIDS, TB and malaria causes the overwhelming number of deaths in the WHO African Region (World Health Organization, 2017). Nevertheless, to date, only HIV/AIDS and malaria RCTs conducted in Africa have been descriptively mapped, while TB has not yet been mapped and appraised (Lutje et al., 2011, Siegfried et al., 2005).”  In addition, several paragraphs provide background regarding the rationale for the conduct of the study to map TB trials. |
| Justify the significance of the study | Thank you.  To clarify and justify the significance and value of the study we have added a paragraph as follows:  “This is the first study using systematic review methods to comprehensively map, describe and evaluate all published RCTs of TB treatment interventions conducted in Africa. We set out to report where trials have been conducted and describe the trial characteristics, including the reporting quality. Thus, our study aims to have highlight potential research and methods gaps in this field of research and inform future clinical research practices and reporting.”  In addition, the background already has the following which adds to the justification considering the value of the results for different role-players.  “In sub-Saharan Africa, where the TB diseases burden is high, it is necessary to understand tested TB treatments and whether they work. This information may help patients and carers, healthcare providers, researchers and policymakers who are required to make decisions regarding best options for TB care. Adequately mapping trial activity presents decision-makers with a comprehensive and instant summary of conducted trials to identify where and what research is happening, and any possible research gaps that need consideration [6]” |
| **Methods** |  |
| What is meant by cross-sectional study for review of studies? Need justification? I think it is miss used | Thank you.  We incorporated systematic review methods to search, screen, and assess the eligibility of published RCTs. Rather synthesising the RCTs, we implemented a cross-sectional approach to describe them and evaluate their quality. Rather than including an analytical component, we added a descriptive component. We extracted data from a pre-defined population and measured the presence or absence of pre-defined characteristics of interest. This definition is found on page 85 of Joubert, G., Ehrlich, R., Katzenellenbogen, J. and Karim, S.A., 2007. *Epidemiology: A research manual for South Africa*. Oxford University Press Southern Africa. |
| Was there a study protocol? | Thank you,  We have an internal protocol for the study, but this was not published.  Furthermore, our methods build on similar studies by Siegfried (2005) that mapped HIV/AIDS trials and Lutje et al. (2011) that mapped malaria trials in Africa.  The following sentence has been added under the heading Material and Methods:  “Our methods build on similar studies that mapped published HIV/AIDS and malaria trials in Africa, respectively [6, 10].” |
| Justify the restrictions placed on for time period (1952 to 2019) and its importance describing 70 years data to gather | Thank you. Our search had no time or language restrictions. 1952 was the year of publication of the first trial for which we found a published RCT.  We have added this explanation to the text as follows:  “We performed a comprehensive search with no restrictions on the language or publication date.” |
| Present the exact search strategy (for at-least one database), with filters used. | The exact search strategy for PubMed is presented in Table 1. |
| How quality is assessed? | We used Cochrane’s risk of bias tool to appraise the quality of included trials.  The following sentence can be found under data extraction:  “Reviewers applied the Cochrane Risk of Bias tool to evaluate methodological quality for the following kinds of bias: selection, detection and performance [16].” |
| State eligibility criteria for inclusion of studies in this review briefly | Thank you. Kindly note that this has already been stated in the methods section of the paper.  “**Inclusion criteria**  All published TB treatment RCTs that included participants with a positive TB diagnosis and had at least one recruitment site in an African country were included.” |
| State how duplicate studies were removed including any software used. | Thank you.  To clarify this, we have amended our current description of the use of Endnote reference management software as follows:  “The reference management software EndNote™ X7 was used to manage the records, including removal of duplicate records.” |
| State how screening of studies was undertaken | Thank you. In the manuscript we outline how screening was done as follows:  “Two authors (AH, LM) did a single screening of all titles and abstracts. AH and LM discussed any unclear records. After that, we retrieved the full-text articles. Two authors (AH, LM) divided the list of full texts for eligibility screening, after which we reviewed each other’s list. Co-authors (TK, EP) were arbiters in cases of disagreement during all phases of screening. The reference management software EndNote™ X7 was used to manage the records, including removal of duplicate records.”  We edited the abstract to make this clear and consistent across the sections of the manuscript. |
| Describe attempts to seek/clarify data from authors of the included studies (if required). | Thank you. We did not come across ambiguous studies that required us to seek clarity from contact authors |
| Describe how missing data were tackled and explain how it was addressed (if any). | Thank you, to clarify the approach to managing missing data, we have added the following explanation to the methods section:  “If data were missing, we reported it as being “not reported”.” |
| **Results** |  |
| PICO is not briefly described | Thank you.  This was previously described under “Inclusion criteria” but has been made clearer as follows:  “All published TB treatment RCTs that included participants with a positive TB diagnosis and had at least one recruitment site in an African country were included.” |
| **Discussion** |  |
| ….. conducted wholly or partly in Africa. What is really done? | Thank you. All trials with a trial site in an African country was eligible to be included. Some trials were multi-national trials with trial sites outside of the African continent.  We have added a sentence to clarify this issue as follows:  “This cross-sectional study aimed to map and describe the clinical and methodological features of published TB intervention RCTs with at least one recruitment site in an African country.” |
| All what is discussed in the discussion section is not describe in the result section. Try to describe | Thank you.  We have reviewed and revised the discussion to ensure alignment with the results and discussion.  If there are further points in the discussion that are not presented in the findings, kindly clarify for us to address. |
| **Reviewer 2:** | Thank you. |
| A more detailed analysis and discussion on subgroups such as MDR-TB, TB-HIV co-infection would be necessary. | Thank you.  Further analysis has now been conducted to determine conditions considered for included by trials.  We have addressed this comment and conduced further analysis of the sub-groups of studies according to MDR-TB, TB-HIV co-infection. This is reported in the results as follows:  “A hundred and four trials (59.43%) included TB patients co-infected with HIV, 56/175 (32%) included TB infected participants alone, 12/175 (6.86%) trials include participants with multi-drug-resistant TB (MDR-TB) co-infected with HIV, 3/175 (1.71%) trials only investigated MDR-TB participants.” |
|  |  |

Thank you for your consideration!

Yours sincerely,

Ameer Steven-Jorg Hohlfeld

MPH (Epid.): University of Cape Town

LUTJE, V., GERRITSEN, A. & SIEGFRIED, N. 2011. Randomized controlled trials of malaria intervention trials in Africa, 1948 to 2007: a descriptive analysis. *Malaria journal,* 10**,** 61.

SIEGFRIED, N., CLARKE, M. & VOLMINK, J. 2005. Randomised controlled trials in Africa of HIV and AIDS: descriptive study and spatial distribution. *Bmj,* 331**,** 742.

WORLD HEALTH ORGANIZATION 2017. 2016 Annual Report Communicable Diseases Cluster.

HIGGINS, J. P. & GREEN, S. 2016. Cochrane handbook for systematic review of interventions version 5.1. 0 [updated March 2011]. The Cochrane Collaboration, 2011. *Available from www. cochrane-handbook. org*.

LUTJE, V., GERRITSEN, A. & SIEGFRIED, N. 2011. Randomized controlled trials of malaria intervention trials in Africa, 1948 to 2007: a descriptive analysis. *Malaria journal,* 10**,** 61.

SIEGFRIED, N., CLARKE, M. & VOLMINK, J. 2005. Randomised controlled trials in Africa of HIV and AIDS: descriptive study and spatial distribution. *Bmj,* 331**,** 742.

WORLD HEALTH ORGANIZATION 2017. 2016 Annual Report Communicable Diseases Cluster.

HIGGINS, J. P. & GREEN, S. 2016. Cochrane handbook for systematic review of interventions version 5.1. 0 [updated March 2011]. The Cochrane Collaboration, 2011. *Available from www. cochrane-handbook. org*.

LUTJE, V., GERRITSEN, A. & SIEGFRIED, N. 2011. Randomized controlled trials of malaria intervention trials in Africa, 1948 to 2007: a descriptive analysis. *Malaria journal,* 10**,** 61.

SIEGFRIED, N., CLARKE, M. & VOLMINK, J. 2005. Randomised controlled trials in Africa of HIV and AIDS: descriptive study and spatial distribution. *Bmj,* 331**,** 742.

WORLD HEALTH ORGANIZATION 2017. 2016 Annual Report Communicable Diseases Cluster.
