# Supplementary material for: Tuberculosis treatment intervention trials in Africa: A cross-sectional bibliographic study and spatial analysis
Source: PLoS One. 2021 Mar 19;16(3):e0248621. doi: 10.1371/journal.pone.0248621 (PMC7978376; doi:10.1371/journal.pone.0248621)
Supplement: Attachment — Submitted filename: Response to Reviewers.docx [file pone.0248621.s005.docx]

Cochrane South Africa

South African Medical Research Council

Francie van Zijl Drive, Parow Valley

Cape Town, 7501

South Africa

22 January 2021

**To: The Editor**

**PLOS ONE**

Dear Editor

Thank you for the feedback. We would like to resubmit the attached manuscript, titled **“Tuberculosis treatment intervention trials in Africa: a cross-sectional bibliographic study and spatial analysis”** after addressing reviewers’ comments. Please see below a table of the reviewers’ comments and our responses.

| **Reviewers’ comments** | **Authors’ response** |
| --- | --- |
| **Abstract** |  |
| In the Conclusions, there is a sentence that is a fragment: "By mapping African TB trials, research gaps." | Thank you for bringing this to my attention.  Wording was erroneously during final editing for submission.  The sentence should read as follows:  “By mapping African TB trials, we were able to identify potential research gaps.” |
| In the Conclusions, there is mention of lead authors from the global north and the lack of child and adolescent participants. However, I cannot see anything in the Results on these topics. Please adjust so that the conclusions have some basis in your findings. | Thank you for bringing this to my attention:  The following edits where made to the Abstract results and conclusion to bring clarity to the reader.  The Abstract’s results sections read as follows:  “First authors were from 30 countries globally. South Africa had the most first authors (n = 55); followed by the United States of America (USA) (n = 28) and Great Britain (n= 14) with fewer other African countries contributing to the first author tally. Children under 13 years of age eligible to participate in the trials made up 17/175 trials (9.71%).”  The Abstract’s conclusion reads as follows:  “Many of the global north’s researchers were found to be the lead authors in these African trials. Few trials tested behavioural interventions compared to drugs, and far fewer tested interventions on children compared to adults to improve TB outcomes.” |
| **Conclusion** | Lastly additional texts were edited in the conclusion section to accurately reflect the results:  “TB treatment trials focused on adults, with far fewer including children.” |

Thank you for your consideration!

Yours sincerely,

Ameer Steven-Jorg Hohlfeld

MPH (Epid.): University of Cape Town
